# Analysis of the Microstructure and Mechanical Properties of Austenitic Stainless Steel 310 Manufactured via WAAM

**DOI:** 10.3390/ma18163855

**Published:** 2025-08-18

**Authors:** Aline Cipriano, Célia de Fraga Malfatti, Henrique Cechinel Casagrande, Anderson Daleffe, Jovani Castelan, Pedro Henrique Menegaro Possamai

**Affiliations:** 1 Department Metallurgical Engineering, Universidade Federal do Rio Grande do Sul (UFRGS), Porto Alegre 90010-150, Brazil; aline.cipriano@satc.edu.br (A.C.); materiaisxenergia@gmail.com (C.d.F.M.); 2 Department Metallurgical Engineering, Universidade Federal de Santa Catarina (UFSC), Florianópolis 88040-900, Brazil; 3 Department Additive Manufacturing, Centro Universitário SATC (UNISATC), Criciúma 88805-380, Brazil; anderson.daleffe@satc.edu.br (A.D.); jovani.castelan@satc.edu.br (J.C.); pedro.possamai@satc.edu.br (P.H.M.P.)

**Keywords:** Wire and Arc Additive Manufacturing (WAAM), AISI 310 austenitic stainless steel, tensile strength and ductility, microstructural characterization, optical emission spectrometry (OES)

## Abstract

The objective of this study was to characterize austenitic stainless steel 310 produced by Wire and Arc Additive Manufacturing (WAAM), addressing a gap in the literature regarding this alloy. Microstructural, chemical, and mechanical analyses were performed. Optical and electron microscopy revealed a predominantly columnar grain structure with characteristic tracks along the deposition direction. Point and mapping EDS analyses indicated a homogeneous distribution of iron, chromium, and nickel; however, point measurements suggested a possible underestimation of nickel, likely due to high relative error. Tensile tests demonstrated anisotropic mechanical behavior, with yield strength meeting standards at 45° and 90°, but lower at 0°. Ultimate tensile strength and elongation were below conventional requirements, with a maximum elongation of 15% at 90°. Additionally, the sample exhibited a total porosity of approximately 0.89%, which contributes to the reduction in mechanical properties, especially in the direction parallel to the deposition tracks. Overall, the WAAM-produced 310 stainless steel presented a microstructure similar to hot-rolled and annealed AISI 310 steel, but with distinctive features related to the additive process, such as mechanical anisotropy and microstructural directionality. These limitations highlight the need for process optimization to improve mechanical performance but reinforce the alloy’s structural potential in additive manufacturing.

## 1. Introduction

Additive manufacturing (AM) represents a modern approach to producing complex components through layer-by-layer construction [1,2,3,4]. AM has the potential to revolutionize the global manufacturing and logistics landscape [5] by offering significantly greater geometric freedom compared to conventional methods such as casting, forging, and machining [6]. A promising example is Wire and Arc Directed Energy Deposition (WA-DED) [7,8,9], also known as Wire and Arc Additive Manufacturing (WAAM) [10,11].

This process has gained attention due to its high deposition rates [12] and suitability for the fabrication of large-scale and geometrically complex components [13,14]. Its operation resembles multipass welding [15], particularly regarding solidification behavior and thermal cycles [16]. According to ASTM (American Society for Testing and Materials), additive manufacturing is defined as a process of material consolidation to create objects from three-dimensional models, typically through layer-by-layer construction, in contrast to subtractive manufacturing techniques [17,18]. The use of metallic materials in AM contributes to cost reduction and environmental impact mitigation by significantly saving material and energy during production [19].

The manufacturing of stainless steels remains a relevant industrial challenge, particularly due to the machining difficulty of these high-value austenitic alloys, caused by their pronounced work hardening and low thermal conductivity [20,21]. Austenitic stainless steels stand out for their unique combination of advantageous properties, such as high strength at both elevated and cryogenic temperatures [22,23,24], rapid strain hardening, excellent ductility, good energy absorption, and strong resistance to corrosion and environmental degradation [25,26]. As a result, they are widely employed across sectors such as petrochemical [27], gas turbines, automotive, medical, civil construction [28], marine, offshore [29], and aerospace industries [30].

These alloys contain higher levels of chromium and nickel compared to conventional ferrous alloys. Their specific chemical composition provides high tensile strength and excellent oxidation resistance, enabling efficient use in applications up to approximately 1323 K (1050 °C) [31]. Numerous studies have investigated the relationship between processing parameters, microstructure, and the properties of austenitic stainless steels, aiming to leverage the economic and productive advantages of WAAM. However, most investigations have focused on the 304, 308, and 316 series [32,33,34,35,36,37,38,39,40,41,42,43,44,45,46,47], whereas the austenitic stainless steel 310, especially concerning its specific microstructural features and mechanical anisotropy, remains underexplored.

The mechanical properties of austenitic stainless steel components produced via WAAM are critical to ensuring reliable performance in safety-sensitive applications. Previous studies have highlighted several factors affecting these properties, including grain structure [48,49,50,51], dendrite geometries [52], δ-ferrite phase presence [53], dislocation density [54,55], inclusions [56], and cracking [57,58,59]. According to Siddiqui et al. [60], other relevant aspects include delamination caused by segregation between adjacent deposited tracks [61] and geometric accuracy [62].

Although some studies have addressed the microstructure and defects such as microcracks and segregations [63], significant gaps remain in the detailed understanding of the correlation between specific deposition conditions, cooling rates, and the anisotropic mechanical behavior of WAAM-fabricated 310 stainless steel. This study differentiates itself by providing an integrated and in-depth investigation of microstructural characterization using both optical and electron microscopy, combined with quantitative analysis of mechanical anisotropy in multiple deposition orientations—areas still scarcely explored in the literature.

Therefore, this study expands the knowledge on the behavior of WAAM-produced 310 stainless steel, providing quantitative data and comparative analyses that support future process improvements and industrial applications of this material, contributing to technological advancement in the additive manufacturing of high-performance stainless steel alloys.

## 2. Experimental Procedure

**Figure 1:**
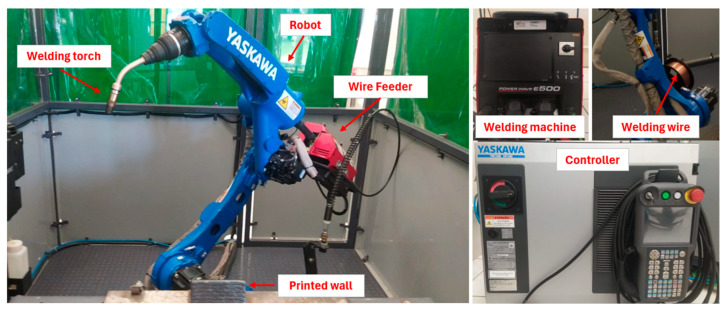
Industrial robot for additive manufacturing.

This study employed the WAAM process to fabricate austenitic stainless steel 310 components. A wall measuring approximately 250 × 130 × 10 (mm) was produced. The feedstock wire, supplied by Superfine, had a diameter of 1.2 mm. The manufacturing system comprised a YASKAWA industrial robot (YASKAWA, São Paulo, Brazil) coupled with a Lincoln Electric welding power source (Lincoln Electric, São Paulo, Brazil). Figure 1 illustrates the equipment setup used in the additive manufacturing process.

After the additive manufacturing of the material, no post-processing treatments or pre-processing steps were performed on the austenitic 310 steel. Optical emission spectrometry testing was conducted for chemical characterization. During deposition, each bead was laid sequentially to form continuous tracks, ensuring proper overlap and consistent layer height throughout the structure.

Wire and Arc Additive Manufacturing (WAAM) of austenitic stainless steel 310 was successfully carried out using carefully selected operating parameters, namely, a voltage of 19 V, a current of 165 A, a wire feed speed of 5.5 m/min, a torch travel speed of 300 mm/min, a shielding gas flow rate of 16 L/min, and a wire diameter of 1.2 mm. These parameters resulted in an approximate power of 3.14 kW and a heat input (Equation (1)) of 627 J/mm, a value considered adequate to ensure proper interlayer fusion and a high deposition rate of approximately 49 g/min (Equation (2)), without compromising the geometric integrity of the built part.

(1) $$\mathrm{Heat}\;\mathrm{Input}\;(J/\mathrm{mm})=\frac{V\times A\times 60}{Torch\;travel\;speed}$$

where

- V = voltage (V).
- A = current (A).
- Travel speed (mm/min).
- Result in joules per millimeter (J/mm).

$$\mathrm{Heat}\;\mathrm{Input}=\frac{19\times 165\times 60}{300}=627\;\frac{J}{mm}$$

Deposition Rate (g/min): m = Area vw⋅ρ(2)

where:

- Area = $\frac{\pi d^{2}\;}{4}$ = cross-sectional area of the wire (mm^2^).
- d = wire diameter (mm).
- vw = wire feed speed (mm/min).
- ρ = density of stainless steel 310 = (7.9 g/cm^3^ or 0.0079 g/mm^3^) [26].
- m = deposition rate (g/min).

m = 49.1 g/min.

The process parameters used for fabricating the austenitic stainless steel 310 wall are presented in Table 1.

As shown in Table 2, the metallic wall was successfully fabricated using Wire and Arc Additive Manufacturing (WAAM), exhibiting an average thickness of 10 mm and a layer height of approximately 2.5 mm, which indicates good dimensional stability and process control throughout the build. A total of 100 successive layers were deposited, resulting in a wall height of approximately 250 mm, consistent with the vertical spacing between layers and the intended geometry. This vertical growth rate per layer (2.5 mm) falls within the expected range for high-deposition-rate processes such as WAAM and reinforces the effectiveness of the selected parameters, particularly in terms of heat input and interlayer fusion.

The average deposition time per bead was 26 s, reflecting a relatively fast deposition rate, a desirable feature in industrial processes aimed at high productivity. The total active fabrication time was only 43 min, demonstrating the potential of the WAAM process for the rapid and efficient production of medium-sized metallic components.

It is worth noting that the relationship between the total manufacturing time and the number of layers suggests that the interpass time was minimal or even nonexistent. This may warrant further microstructural evaluation, as consecutive thermal cycles can influence columnar grain growth and the formation of undesirable phases in highly alloyed materials such as 310 stainless steel.

Overall, the results show that the applied parameters enabled efficient construction, with adequate dimensional control, high deposition rate, and low production time—characteristics that highlight WAAM as a viable alternative for fabricating structural components using corrosion- and heat-resistant stainless steels.

**Figure 2:**
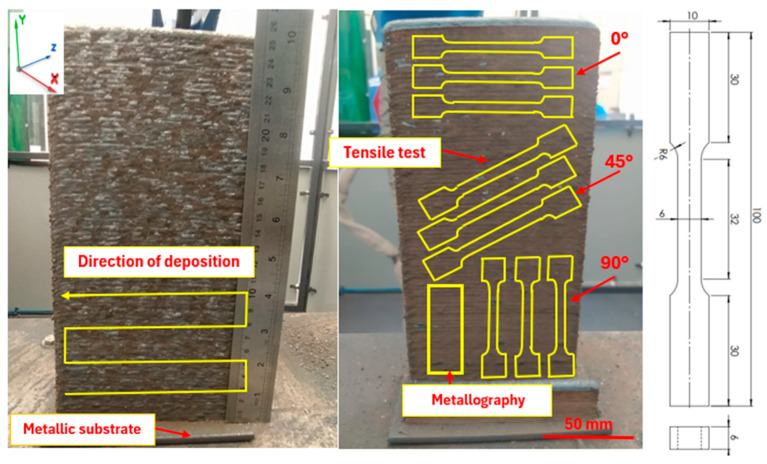
Wall printed using the Wire and Arc Additive Manufacturing (WAAM) process with 310 stainless steel.

Figure 2 depicts the wall fabricated by additive manufacturing using a zig-zag bead deposition strategy. The image also presents the tensile test specimens prepared for anisotropy analysis, along with a sample designated for metallographic examination. The sample detailed in Figure 2 for mechanical tensile testing follows the ASTM E8 standard [64].

Subsequently, the edges of the wall were symmetrically trimmed, leaving a remaining structure measuring 120 mm in length. This was performed to remove the inherent surface waviness caused by the WAAM bead step effect [65].

The material used as the substrate was a 310 stainless steel plate with a thickness of 6.14 mm, 75 mm in width, and 150 mm in length. During the additive manufacturing process, the deposition torch was positioned at 90° relative to the track deposition direction (*Y*-axis).

### 2.1. Microstructural Characterization

Scanning electron microscopy (SEM) and energy dispersive spectroscopy (EDS) analyses were performed using a Zeiss EVO MA 10 microscope (São Paulo, Brazil) operated at 10 kV with a tungsten filament. To reveal the phases present in the samples, Vilella’s reagent was applied for 180 s for both SEM and optical microscopy (OM) analyses. Optical microscopy (OM) was conducted using an Olympus SC30 microscope (São Paulo, Brazil). The samples were sectioned using a metallographic cutter, brand Struers (Milton, Australia), model MESOTOM. Subsequently, the samples were extracted along the deposition track and underwent grinding with different grit sizes (80, 120, 200, 320, 400, 600, and 1200). After grinding, the samples were polished using a polisher, brand Fortel (Willenhall, UK), model PFL, with Fortel alumina polishing suspension of 1-micron particle size diluted in water.

### 2.2. Tensile Testing

Tensile tests were conducted at a crosshead speed of 5 mm/min using an InterMetric GR173 testing machine (São Paulo, Brazil) with a maximum capacity of 200 kN. The specimens to be tested were machined using a ROMI U30 milling machine (São Paulo, Brazil). After milling, to reduce the surface roughness, the specimens were subjected to grinding using a Mello P36 surface grinder (São Paulo, Brazil). Following machining, the specimens were cut according to the ASTM E8 standard using an AMADA FLC 3015 AJ laser cutting machine (Kanagawa, Japan). To evaluate the anisotropy of the WAAM-processed material, tensile specimens were extracted in three orientations relative to the deposition track direction: 0°, 45°, and 90°. For each orientation, the tests were performed in triplicate to ensure statistical reliability of the mechanical behavior assessment.

### 2.3. Optical Emission Spectrometry (OES)

For the optical emission spectrometry (OES) analysis, a BRUKER Q2 ION model spectrometer (Billerica, MA, USA) was used, operating at a power of 400 Watts for 30 s. The sample was initially sectioned using a metallographic cutter, brand Struers, model MESOTOM. Subsequently, it was machined using a Romi U30 milling machine (São Paulo, Brazil) and then ground with a Mello P36 surface grinder (São Paulo, Brazil) in order to reduce surface roughness.

### 2.4. Characterization of Pore Distribution

The sample was initially prepared using a metallographic cutter from Struers, model MESOTOM. The samples underwent a progressive grinding process using abrasive papers with grit sizes of 80, 120, 200, 320, 400, 600, and 1200. Subsequently, the samples were polished using a polisher from Fortel, model PFL, employing a Fortel alumina polishing suspension with 1-micron particle size diluted in water. After the surface preparation, the sample was analyzed using an Olympus SC30 optical microscope (São Paulo, Brazil), without chemical etching, aiming exclusively to reveal the presence of pores. The quantification of porosity was performed using the software PRECiV Pro 1.2, which enabled the determination of the distribution and amount of pores in the sample.

## 3. Results and Discussion

### 3.1. Metallographic Analysis Using an Optical Microscope on 310 Austenitic Steel

**Figure 3:**
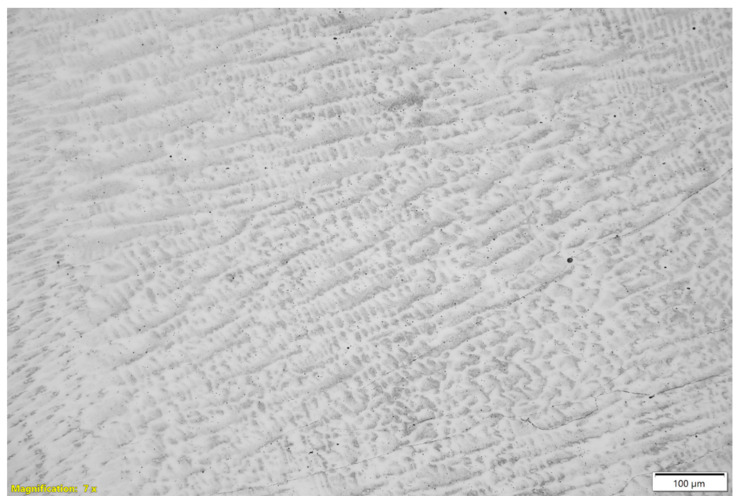
Metallographic analysis of 310 austenitic stainless steel produced by Wire and Arc Additive Manufacturing (WAAM) at 200× magnification.

**Figure 4:**
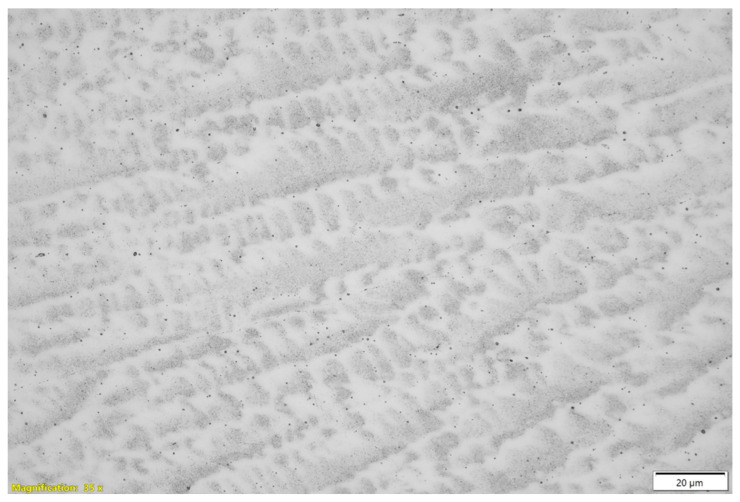
Metallographic analysis of 310 austenitic stainless steel produced by Wire and Arc Additive Manufacturing (WAAM) at 1000× magnification.

The columnar grain growth observed in Figure 3 and Figure 4 is typical of arc-based deposition processes, due to the strong thermal gradient and the preferential solidification direction [1]. This microstructure can induce anisotropy in the mechanical properties, as supported by the tensile test results presented. The presence of grain boundary segregations, suggested by contrast variations and EDS analyses, may negatively affect the corrosion resistance and ductility of the material, as reported by [66,67].

Compared to other studies involving austenitic stainless steel fabricated by the WAAM process [68,69,70], our results confirm that the thermal and cooling conditions during deposition are decisive for the resulting microstructure, highlighting the importance of precise control of welding parameters to optimize component properties. In particular, recent studies estimate that the cooling rates during WAAM of AISI 310 stainless steel can range from approximately 200 to 1130 K/s, depending on the process parameters and geometry, which is consistent with the refined and columnar dendritic structures observed in the present study [1].

More specifically, microstructural analyses using optical microscopy and scanning electron microscopy, focusing on the interlayer regions, revealed features that contribute to the observed anisotropic mechanical behavior. These include localized porosity and micropores along the interfaces between successive layers, partial lack of fusion between adjacent weld beads, interlayer oxide films detected by compositional contrast and EDS, localized microsegregation of elements, especially nickel depletion, and discontinuities in dendritic grain boundaries formed during directional solidification. Additionally, the relatively uniform size of the columnar grains directly influences the observed ductile fracture mode, promoting energy absorption and the formation of dimples on the fracture surface. These interfacial and microstructural features act as critical points for crack initiation and propagation, weakening mechanical cohesion and explaining the reduction in tensile strength and ductility in samples tested parallel to the deposition direction.

### 3.2. Scanning Microscope Metallographic Analysis of 310 Austenitic Steel

**Figure 5:**
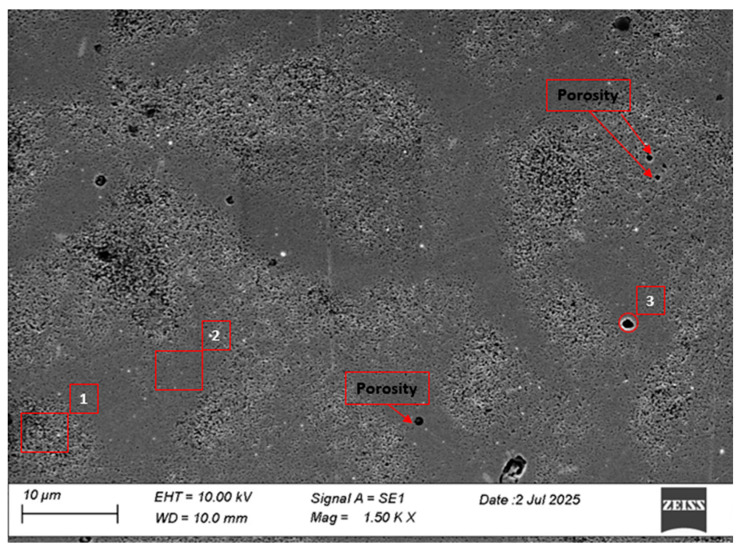
SEM metallographic analysis of 310 austenitic stainless steel fabricated by Wire and Arc Additive Manufacturing (WAAM).

The SEM micrograph of AISI 310 stainless steel produced by WAAM, presented in Figure 5, reveals a relatively uniform matrix with the presence of microporosity and localized granular agglomerates. These features are typical of the overlapping thermal cycles and rapid solidification conditions inherent to arc-based additive manufacturing processes. The presence of porosity and microstructural heterogeneity may compromise the mechanical performance and durability of the component, particularly in corrosive environments. Previous studies have shown that reducing porosity is closely associated with the optimization of welding parameters, such as current, deposition speed, and bead overlap [71].

**Figure 6:**
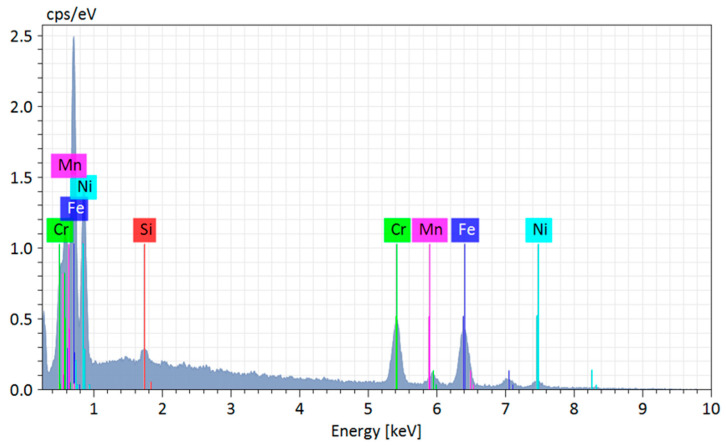
EDS analysis at point 1 for 310 austenitic stainless steel produced by Wire and Arc Additive Manufacturing (WAAM).

The EDS analysis performed at Point 1 (Figure 6), located in a visually homogeneous region of the matrix, identified the elements Fe (iron), Cr (chromium), Ni (nickel), Mn (manganese), and Si (silicon), all consistent with the nominal composition of 310 stainless steel. The prominent peaks of Fe, Cr, and Ni confirm that the analyzed matrix is predominantly an austenitic solid solution. However, the presence of Si at detectable levels, although expected (as silicon acts as a deoxidizer and ferrite stabilizer), may also suggest a certain degree of segregation during solidification. This behavior has been previously reported in studies on stainless steels produced by WAAM, where thermal gradients and constant reheating promote element partitioning between dendritic regions or along grain boundaries [63,72,73,74]. Although the analyzed point was not directly located within such segregated zones, the slight increase in silicon and manganese suggests that even apparently homogeneous areas may exhibit chemical composition fluctuations, which can affect localized corrosion resistance and mechanical performance.

**Figure 7:**
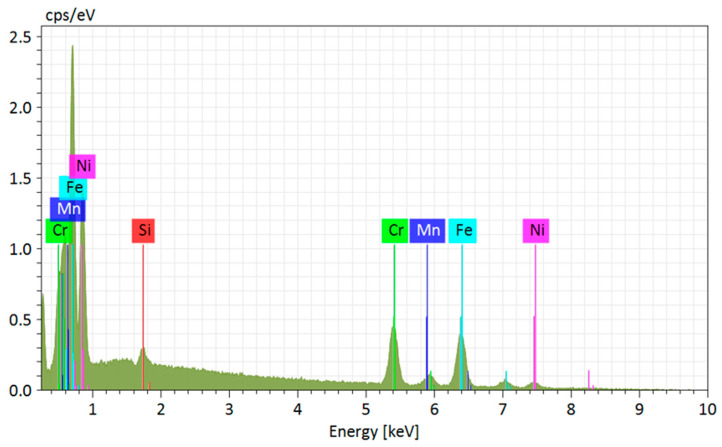
EDS analysis at point 2 for austenitic 310 stainless steel fabricated by Wire and Arc Additive Manufacturing (WAAM).

The point EDS analysis conducted at Region 2 (Figure 7) revealed the presence of key alloying elements characteristic of 310 stainless steel, such as iron (Fe), chromium (Cr), and nickel (Ni), as well as manganese (Mn) and silicon (Si). The high Cr and Ni contents are essential for stabilizing the austenitic phase and ensuring oxidation and corrosion resistance at elevated temperatures [26,53]. The detection of Si, albeit at lower intensity, may be associated with the use of deoxidizing elements during wire production or with the formation of surface silicon oxide inclusions, which may be confirmed through elemental mapping analyses. The observed surface texture, with contrast variations, indicates potential microsegregation of alloying elements, especially Cr and Ni, among different solidification regions. According to Scudino et al. [75] and Jin et al. [76], this behavior is typical of WAAM processes, where multiple thermal cycles promote elemental redistribution and the formation of zones with distinct metallographic morphologies.

**Figure 8:**
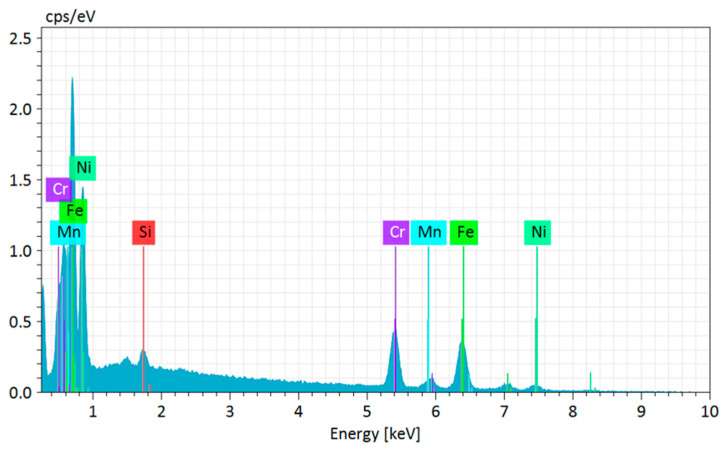
EDS analysis at point 3 for austenitic 310 stainless steel produced by Wire and Arc Additive Manufacturing (WAAM).

Figure 8 presents the EDS analysis corresponding to Point 3. While Fe and Cr were again identified as the predominant elements consistent with the austenitic matrix of the alloy, a significant reduction in Ni concentration was observed. This discrepancy may be attributed to the low excitation efficiency of Ni peaks under the analysis conditions, the local surface topography, or more likely, to microsegregation or local nickel depletion during solidification. Such behavior has been reported by Keller et al. [77] and Nedjad et al. [78], who observed localized Ni depletion in specific regions of austenitic stainless steels deposited by WAAM, with direct implications for phase stability. Despite the uncertainty in Ni quantification at Point 3, the detection of Mn and Si in typical proportions suggests that the austenitic matrix was generally preserved. Nevertheless, the chemical heterogeneity observed, particularly in Ni distribution, may compromise intergranular corrosion resistance and negatively affect local mechanical properties. These variations highlight the need for complementary strategies, such as post-deposition heat treatments and optimization of welding parameters, to reduce interlayer chemical gradients and promote greater microstructural uniformity [79,80]. Therefore, the results obtained are consistent with several studies in the literature that describe the metallurgical challenges associated with the fabrication of stainless steels via the WAAM process. The observations confirm the presence of a typical austenitic microstructure; however, local compositional variations and process-inherent defects such as porosity and segregation were also identified. These factors must be carefully considered during the design and qualification of critical components manufactured through Wire and Arc Additive Manufacturing.

**Figure 9:**
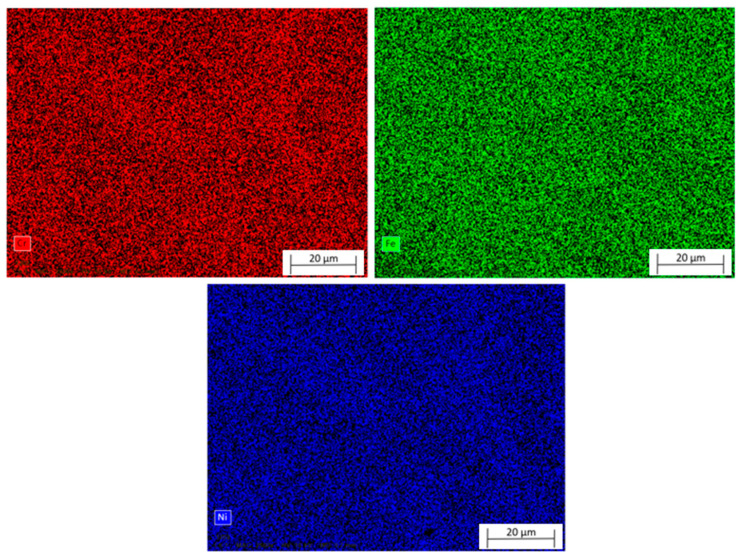
Scanning electron microscopy analysis of austenitic 310 stainless steel fabricated by Wire and Arc Additive Manufacturing (WAAM).

Figure 9 presents the elemental mapping obtained by energy-dispersive X-ray spectroscopy (EDS) of the surface of AISI 310 austenitic stainless steel produced by Wire and Arc Additive Manufacturing (WAAM), highlighting the distribution of chromium (Cr), iron (Fe), and nickel (Ni). The analysis reveals a homogeneous distribution of the main alloying elements within the mapped region, indicating good chemical uniformity at the micrometric scale. Chromium, a key element in the formation of the passive film and, consequently, in corrosion resistance, was uniformly dispersed across the analyzed area. This suggests the absence of chromium-depleted zones, which could otherwise compromise resistance to localized corrosion. Iron, the primary matrix element, also displayed a uniform distribution, supporting the structural stability of the alloy. Although the nickel mapping showed slight intensity variations, no significant segregation or localized accumulation of the element was observed. This observation aligns with the point EDS results, in which nickel quantification yielded slightly underestimated values in some regions; however, when viewed along with the mapping data, these deviations appear to result from local analytical limitations rather than actual microsegregation.

The observed compositional homogeneity indicates that, despite the intrinsic anisotropies of the WAAM process, such as columnar textures or interlayer thermal gradients, the deposition process ensured effective fusion and mixing between adjacent weld beads. Similar findings were reported by Rahimi et al. [63,76], who demonstrated that, despite the thermal complexities inherent to WAAM, the distribution of Cr and Ni remains stable when process parameters are properly optimized. Therefore, the EDS mapping results confirm the preservation of the austenitic matrix and the overall chemical uniformity of the material, both of which are critical for the mechanical performance and corrosion resistance of WAAM-deposited 310 stainless steel.

### 3.3. Mapping of Porosity in the Sample

**Figure 10:**
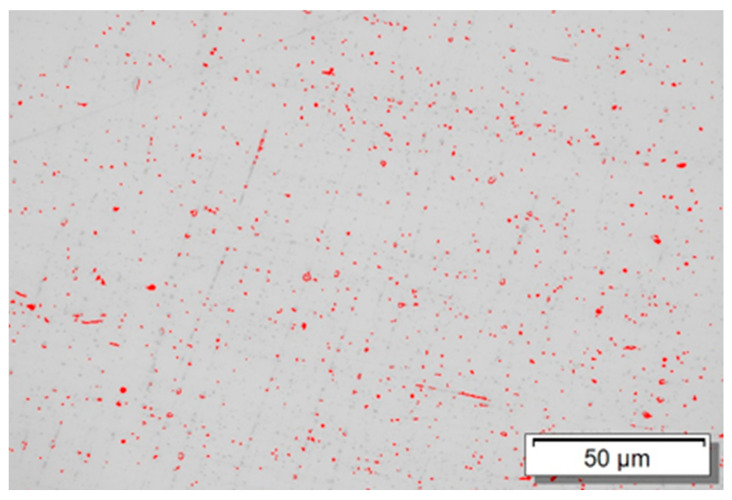
Porosity distribution in the sample produced by Wire and Arc Additive Manufacturing (WAAM).

Quantitative analysis of the micrograph revealed a total porosity of approximately 0.89% in the AISI 310 stainless steel sample produced by WAAM. Although this value falls within the range commonly reported for Wire and Arc Additive Manufacturing processes, its presence is not negligible and can significantly influence the mechanical behavior of the material. The pores act as stress concentrators and potential crack initiation sites, especially under tensile loading, contributing to the reduction in ductility and tensile strength. This influence is particularly relevant in the longitudinal direction (0°), where tensile test results showed the lowest elongation (12.5%) and lowest yield strength (198 MPa). The non-uniform distribution of pores along the deposited layers can also intensify the anisotropic characteristics observed, as pore morphology, orientation, and position relative to interlayer boundaries affect each loading direction differently. Figure 10 illustrates the distribution of pores in the analyzed region, reinforcing the microstructural heterogeneity introduced by the WAAM process. Therefore, the porosity identified in this study can be considered a contributing factor to the localized degradation of mechanical properties and the typical anisotropy found in WAAM-produced components.

### 3.4. Optical Emission Spectrometry for the Chemical Characterization of AISI 310 Stainless Steel

The chemical composition of the AISI 310 stainless steel sample was determined by optical emission spectrometry (OES), as presented in Table 3. The results confirm typical contents for this type of steel, with approximately 26.70 wt.% Cr, 20.10 wt.% Ni, 1.32 wt.% Mn, and the remainder predominantly composed of Fe (51.14 wt.%), in addition to minor fractions of Si (0.476 wt.%), Mo (0.14 wt.%), and C (0.12 wt.%). This analysis was carried out to quantitatively and reliably validate the data obtained from energy-dispersive X-ray spectroscopy (EDS), which, due to its intrinsic limitations, may lack quantitative accuracy, particularly for light or low-concentration elements such as nickel (Ni) and carbon (C).

In the specific case of Point 3 in the EDS analysis (Figure 8), although the austenitic matrix was confirmed by the predominance of Fe and Cr, a significant reduction in Ni content was observed. This discrepancy may be attributed to inefficient excitation of Ni peaks under EDS conditions, to local surface topography, or more likely, to microsegregation or localized nickel depletion during solidification, as previously reported in the literature (Keller et al. [77]; Nedjad et al. [78]). When these EDS results are compared to those obtained via OES, which indicate a bulk Ni content of approximately 20.10 wt.%, in line with AISI 310 specifications, it is possible to infer that the overall chemical matrix is preserved, while the local variations detected by EDS reflect the layer-wise deposition characteristics of the WAAM process, such as thermal gradients and partial remelting between adjacent tracks.

### 3.5. Mechanical Tensile Test on 310 Austenitic Steel

**Figure 11:**
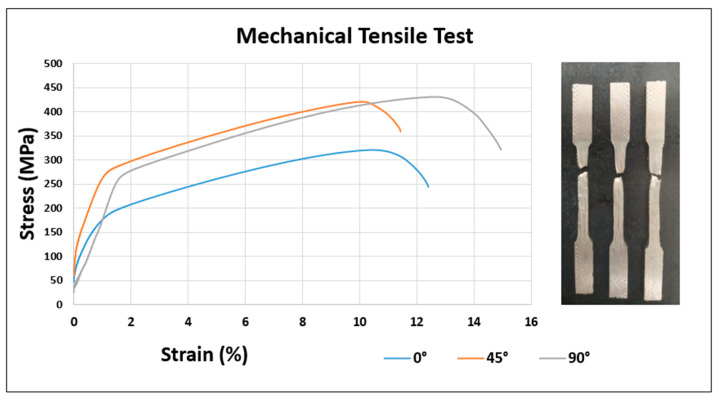
Mechanical tensile test at 0°, 45°, and 90° of specimens fabricated by Wire and Arc Additive Manufacturing (WAAM).

Figure 11 presents the tensile test results for AISI 310 austenitic stainless steel specimens fabricated via WAAM, tested in three orientations relative to the deposition direction: 0°, 45°, and 90°. The values shown correspond to the average of three specimens per condition, highlighting the anisotropic mechanical behavior typically observed in components produced by layer-by-layer additive manufacturing processes [81,82]. Samples tested at 0°, parallel to the deposition direction, exhibited the lowest tensile strength, with average values around 320 MPa and reduced final elongation, suggesting that the interfaces between successive layers act as weak zones.

Microstructural analyses performed by optical microscopy (OM) and scanning electron microscopy (SEM), focused on the interlayer regions, revealed features that explain this fragility, including localized porosity and micropores, partial lack of fusion between adjacent beads, and localized elemental microsegregation, especially of nickel. Additionally, discontinuities were observed in the grain boundaries of the dendritic columns formed during directional solidification, constituting preferential sites for crack initiation and propagation. These microstructural particularities act as critical points that compromise mechanical cohesion and explain the lower strength and ductility in specimens tested parallel to the deposition direction.

This behavior is widely reported in the literature for metallic alloys produced by WAAM, where lack of fusion or interlayer oxidation can compromise longitudinal cohesion [83,84]. Conversely, specimens tested at 90°, perpendicular to the deposition direction, reached higher tensile strength values (up to 430 MPa) and exhibited elongations exceeding 15%, indicating greater structural integrity in the transverse direction. This improved performance is attributed to the morphology of dendritic columns formed during directional solidification, which, in this orientation, provides greater resistance to crack propagation [85].

Specimens oriented at 45° showed intermediate mechanical behavior in terms of both ultimate tensile strength and elongation. This gradient of properties reinforces the importance of loading orientation in WAAM-fabricated structural components, as mechanical anisotropy can impact the predictability of in-service performance. Despite the inherent ductility of 310 stainless steel, the results clearly demonstrate that the final mechanical performance is significantly influenced by the manufacturing direction. Similar findings have been reported by Wang et al. [83], Rodriguez et al. [86], and Laghi et al. [87], who showed that tensile strength and toughness can vary depending on the testing direction in WAAM-produced parts. Therefore, for critical applications, especially those subjected to multiaxial loading, proper control of component orientation relative to the load direction is essential.

Table 4 summarizes the mechanical behavior of the WAAM-produced AISI 310 stainless steel in three different specimen orientations (0°, 45°, and 90°) relative to the deposition direction. A significant anisotropy is observed in the mechanical properties. The highest yield strength (280 ± 5.13 MPa) was obtained at 45°, while the lowest (198 ± 7.20 MPa) occurred at 0°, which is below the minimum standard requirement of 205 MPa for AISI 310 [88]. Ultimate tensile strength also varied with orientation, reaching a maximum of 430 ± 12.06 MPa at 90°, but remaining below the nominal requirement of 520 MPa in all positions. Elongation followed a similar trend, with the highest ductility (15 ± 1.69%) in the 90° direction, although still significantly lower than the expected 40%. The standard deviations observed, particularly in tensile strength and elongation, reflect the inherent variability of the WAAM process and further support the evidence of anisotropy. These results highlight the directional dependence of the mechanical performance in WAAM-produced components, likely due to thermal cycling and microstructural variations inherent to the process.

To provide additional context and support a broader comparison, Table 5 presents tensile test results from previous studies involving different austenitic stainless steels fabricated by WAAM, including alloys 304, 308L, and 316L. These data help frame the performance of WAAM AISI 310 within the existing literature and emphasize the need to understand alloy-specific responses and anisotropy patterns.

Compared to the literature summarized in Table 5, the mechanical properties of the WAAM-produced AISI 310 stainless steel in this study are generally lower, especially in terms of elongation and ultimate tensile strength (UTS). While other austenitic stainless steels such as 316L and 308L exhibit elongation values above 35% and UTS exceeding 540 MPa, the 310 alloy produced via WAAM in this study achieved a maximum elongation of only 15 ± 1.69% and a UTS of 430 ± 12.06 MPa (in the vertical orientation).

Additionally, when comparing these results to the standard values reported for laminated sheet and forged AISI 310 (ASM Handbook, 1990) [92], a similar trend is observed. The sheet-processed AISI 310 exhibits yield strength (YS) of 240 MPa, UTS of 570–600 MPa, and elongation of 40–46%, while the forged version of 310L shows even higher ductility, with elongation reaching 54%. In contrast, the WAAM-fabricated samples presented YS ranging from 198 ± 7.20 MPa (0°) to 280 ± 5.13 MPa (45°) and elongation between 11.5 ± 2.72% (45°) and 15 ± 1.69% (90°).

This discrepancy may be related to several microstructural and processing factors, including the presence of pores detected in the microstructural analysis, interlayer interfaces, microstructural heterogeneities, and potential elemental segregation during solidification. Furthermore, the limited and discontinuous presence of δ-ferrite, combined with clean γ-dendritic grain boundaries, may contribute to localized ductility loss in specific orientations. These results highlight the influence of alloy-specific solidification behavior and thermal cycling in the WAAM process on the anisotropic mechanical response of the components, indicating the need for further process optimization in the case of AISI 310.

## 4. Conclusions

Based on the results obtained in this study, it is concluded that the austenitic stainless steel 310 manufactured via Wire and Arc Additive Manufacturing (WAAM) exhibits a microstructure characteristic of directional solidification, with prominent columnar grain morphology, dispersed porosity, and visible layer overlap marks. Energy-dispersive X-ray spectroscopy (EDS), through point and mapping analyses, confirmed a global chemical composition consistent with the standard alloy, showing a homogeneous distribution of Fe, Cr, and Ni. However, a slight underestimation of nickel content was observed in specific regions, likely due to local limitations of the point analysis, which highlights the importance of mapping for a more representative and accurate compositional assessment. Optical emission spectrometry (OES) was employed for quantitative validation of the overall chemical composition, confirming a nickel content around 20.10 wt.%, in agreement with the nominal composition of AISI 310 stainless steel. This complementary analysis reinforces the reliability of the chemical characterization and the preservation of the austenitic matrix, despite the local fluctuations detected by EDS.

Tensile tests revealed anisotropic mechanical behavior, with lower strength and elongation in samples extracted parallel to the deposition direction (0°), attributed to the presence of interlayer interfaces, which may be weakened by lack of fusion or porosity. Specifically, specimens tested at 0° presented the lowest yield strength (198 ± 7.20 MPa), ultimate tensile strength (320 ± 4.16 MPa), and elongation (12.5 ± 0.98%). In contrast, the best mechanical performance was observed at 90°, with higher values of yield strength (269 ± 10.21 MPa), tensile strength (430 ± 12.06 MPa), and ductility (15 ± 1.69%). These values, along with the obtained standard deviations, indicate not only the directional dependence of mechanical properties but also the variability inherent to the WAAM process. The highest yield strength (280 ± 5.13 MPa) was unexpectedly observed at 45°, suggesting a complex interaction between grain orientation and stress direction. This behavior is consistent with the literature reports describing the influence of columnar grain orientation and the nature of layer interfaces on the final mechanical response of components produced by Wire and Arc Additive Manufacturing (WAAM).

Furthermore, the metallographic analysis performed without chemical etching allowed the exclusive revelation of pores in the sample. The porosity quantification showed a total value of approximately 0.89%. This porosity directly impacts the tensile test results, especially in samples with orientation parallel to the deposition, contributing to the reduction in mechanical properties due to the presence of internal discontinuities.

Overall, the results confirm that AISI 310 stainless steel manufactured by Wire and Arc Additive Manufacturing (WAAM) maintains chemical composition and mechanical performance suitable for industrial applications, provided that its specific microstructural features and mechanical anisotropy are properly considered in the design and structural assessment of components subjected to multidirectional loading. The potential development of residual stresses inherent to the WAAM process and their implications for dimensional stability are also acknowledged and warrant further investigation. It is recommended to perform X-ray diffraction (XRD) analyses for precise determination of the phases and crystallographic structures present, as well as accelerated corrosion tests to evaluate the material’s resistance under severe service conditions, aiming for a more comprehensive understanding of the long-term performance of AISI 310 stainless steel components produced by WAAM.

When comparing the results obtained in this study with the values presented in Table 5, it is observed that the mechanical properties of the 310 stainless steel produced by WAAM were lower than those reported for similar austenitic alloys fabricated by the same process, such as 316L and 308L, as well as lower than the properties of 310 steels processed by conventional routes, such as rolling and forging. In particular, the ultimate tensile strength and ductility showed reduced values, with a maximum elongation of only 15% in the 90° orientation. This difference can be attributed to the presence of interlayer porosity, weak interfaces between deposited layers, microstructural heterogeneities, and possible elemental segregation during solidification, which negatively affect mechanical performance. A limitation of the present study is the absence of corrosion resistance and high-temperature performance analyses, which are essential aspects for the industrial application of the material. Therefore, future investigations should prioritize these topics to broaden the understanding of the durability and reliability of 310 stainless steel produced by Wire and Arc Additive Manufacturing.

## Figures and Tables

**Table 1:** Parameters used for the additive manufacturing of austenitic stainless steel 310.

Variables	Parameters
Voltage (V)	19
Current (A)	165
Gas flow rate (L/min)	16
Wire feed speed (m/min)	5.5
Power (kW)	3.135
CNC travel speed (mm/min)	300
Wire diameter (mm)	1.2

**Table 2:** Dimensions of the wall fabricated by additive manufacturing.

Variables	Values
Average thickness (mm)	10
Average layer height (mm)	2.5
Number of deposited layers	100
Deposition time per bead (S)	26
Total active fabrication time (min)	43
Shielding gas argon (%)	100

**Table 3:** Chemical composition of the part made of AISI 310 stainless steel.

Elements	Values
Carbon (C)	0.120
Silicon (Si)	0.476
Manganese (Mn)	1.320
Chromium (Cr)	26.70
Nickel (Ni)	20.10
Molybdenum (Mo)	0.140
Iron (Fe)	51.144

**Table 4:** Tensile test results.

Variables: Position	0°	45°	90°
Yield strength (MPa)	198 ± 7.20	280 ± 5.13	269 ± 10.21
Ultimate tensile strength (MPa)	320 ± 4.16	419 ± 12.66	430 ± 12.06
Strain (%)	12.5 ± 0.98	11.5 ± 2.72	15 ± 1.69

**Table 5:** Tensile test results for different austenitic stainless steels produced by WAAM.

Reference	Alloy	Tested Orientations	Ys (MPa)	UTS (MPa)	Elongation (%)
Gowthaman et al., 2023 [89] (WAAM)	316L	Horizontal (0°)	302	576	35
		Vertical (90°)	295	469	39
Anand et al., 2023 [90] (WAAM)	308L	Horizontal (0°)	364	548	38
				
Rani et al., 2022 [91] (WAAM)	304	Horizontal (0°)	-	600	-
		Vertical (90°)	-	597	-
ASM Handbook 1990 [92] (Sheet)	310	Horizontal (0°)	240	570	40
		Vertical (90°)	240	600	46
ASM Handbook 1990 [92] (Forging)	310L	Vertical (90°)	260	585	54
				

## Data Availability

The original contributions presented in this study are included in the article. Further inquiries can be directed to the corresponding author.

## References

[1] Rahimi A., Yazdizadeh M., Ara M.V., Pouranvari M. (2025). CMT wire-arc additive manufacturing of 310 austenitic stainless steel: Microstructure-properties relationships. J. Mater. Res. Technol..

[2] Moreira É.A., Bomfim M.H.S., Liberato F.M. (2005). A evolução da fabricação: Comparação e sinergia entre manufatura aditiva e subtrativa. Res. Soc. Dev..

[3] Cunningham C.R., Flynn J.M., Shokrani A., Dhokia V., Newman S.T. (2018). Invited review article: Strategies and processes for high quality wire arc additive manufacturing. Addit. Manuf..

[4] Zhang T., Li H., Gong H., Wu H., Ahmad A.S., Chen X. (2021). Effect of rolling force on tensile properties of additively manufactured Inconel 718 at ambient and elevated temperatures. J. Alloys Compd..

[5] Frazier W.E. (2014). Metal Additive Manufacturing: A Review. J. Mater. Eng. Perform..

[6] Lee J., Lee C., Kim D. (2022). Repair of damaged parts using wire arc additive manufacturing in machine tools. J. Mater. Res. Technol..

[7] Jeffus L.F. (1997). Welding and Applications.

[8] Zhang C., Li Y., Gao M., Zeng X. (2018). Wire arc additive manufacturing of Al-6Mg alloy using variable polarity cold metal transfer arc as power source. Mater. Sci. Eng. A.

[9] Rodrigues T.A., Duarte V., Miranda R.M., Santos T.G., Oliveira J. (2019). Current Status and Perspectives on Wire and Arc Additive Manufacturing (WAAM). Materials.

[10] Campatelli G., Montevecchi F., Venturini G., Ingarao G., Priarone P.C. (2020). Integrated WAAM-subtractive versus pure subtractive manufacturing approaches: An energy efficiency comparison. Int. J. Precis. Eng. Manuf. Technol..

[11] Tabernero I., Paskuala A., Álvarezb P., Suárezcb A. Study on Arc Welding processes for High Deposition Rate Additive Manufacturin. Proceedings of the CIRP Conference on Electro Physical and Chemical Machinin.

[12] Yan L., Chen Y., Liou F. (2020). Additive manufacturing of functionally graded metallic materials using laser metal deposition. Addit. Manuf..

[13] Ferreira C., Schaeffer L., Daleffe A., Castelan J., Casagrande H., March G.M.A. (2025). Hybrid Manufacturing Process and Hot Forging: Preform Manufacturing Through Localized Melting Material Deposition Using UTP AF DUR 600 Wire. RGSA J. Soc. Environ. Manag..

[14] Casagrande H.C., Daleffe A., Ferreira C.A., Milanez A., March G., Schaeffer L. (2025). Additive Manufacturing in the Forging Process: Preform Manufacturing Through Localized Melting Material Deposition Using Low Carbon Wire. RGSA J. Soc. Environ. Manag..

[15] Casagrande H.C., Daleffe A., Ferreira C., Fritzen D., March G., Castelan J. (2023). Processo de fabricação de peças metálicas por manufatura aditiva com fusão localizada de aços baixa liga. Open Sci. Res..

[16] Li K., Chen W., Gong N., Pu H., Luo J., Zhang D.Z., Murr L.E. (2023). A critical review on wire-arc directed energy deposition of high-performance steels. J. Mater. Res. Tecnol..

[17] Kumar S.A., Prasad R.V.S. (2021). Chapter 2—Basic Principles of Additive Manufacturing: Different Additive Manufacturing Technologies. Additive Manufacturing. A Tool for Industrial Revolution 4.0.

[18] (2012). Standard Terminology for Aditive Manufacturing Technologies.

[19] Uralde V., Veiga F., Aldalur E., Suarez A., Ballesteros T. (2022). Symmetry and Its Application in Metal Additive Manufacturing (MAM). Symmetry.

[20] Korkut I., Kasap M., Ciftci I., Seker U. (2004). Determination of optimum cutting parameters during machining of AISI 304 austenitic stainless steel. Mater. Des..

[21] Pixer F., Arnoldt A., Unger M., Scneider-Broskamp C., Bharadwaj K., Mayrhofer F., Gradinger R., Klein T. (2025). On the viability of in-situ alloying via process gas mixtures in wire arc directed energy deposition of austenitic stainless steel. J. Mech. Work. Technol..

[22] Kou S. (2003). Welding Metallurgy.

[23] Lippold J.C. (2015). Welding Metallurgy and Weldability.

[24] Li J., Yi M., Wu H., Fang Q., Liu Y., Liu B., Zhou K., Liaw P.K. (2020). Fine-grain-embedded dislocation-cell structures for high strength and ductility in additively manufactured steels. Mater. Sci. Eng. A.

[25] Gardner L., Talja A., Baddoo N.R. (2006). Structural design of high-strength austenitic stainless steel. Thin-Walled Struct..

[26] (1994). ASM Specialty Handbook: Stainless Steels.

[27] Sousa J.M.B.A. (2021). Influência dos elementos de liga nos aços inoxidáveis duplex e superduplex utilizados na indústria do petróleo: Uma revisão. Trabalho de Conclusão de Curso apresentado ao curso de Engenharia Metalúrgica do Departamento de Engenharia Metalúrgica e de Materiais da Universidade Federal do Ceará. https://repositorio.ufc.br/bitstream/riufc/60634/3/2021_tcc_jmbasousa.pdf.

[28] Souza V.P., Labiapari W.S., Lins V.F.C. (2024). Stainless steels as a solution for corrosion and erosion problems involving grains in agribusiness sector applications. J. Mater. Res. Technol..

[29] Yadav A., Srivastava M., Jain K. (2025). Design and fabrication of wire arc additive manufacturing setup and enhanced tailored properties of dissimilar steel additively deposited by WAAM process. Estruturas.

[30] Blakey-Milner B., Gradl P., Snedden G., Brooks M., Pitot J., Lopez E., Leary M., Berto F., Plessis A. (2021). Metal additive manufacturing in aerospace: A review. Mater. Des..

[31] Kanni R.A. (2013). On High-Temperature Matersials: A Case on Creep and Oxidation of a Fully Austenitic Heat-Resistant Super-alloy Stainless Steel Sheet. J. Mater..

[32] Liao H., Wang Z., Chi P., Zhang B., Ding T., Zhangc Q. (2024). Evolutions of microstructure and mechanical property of 308L stainless steel repaired by the local dry underwater wire arc additive manufacturing. Mater. Sci. Eng. A.

[33] Chamim M., Darmadi D.B., Purnowidodo A., Widodo T.D., Ismail Z. (2024). Influence of the welding thermal cycle on δ-ferrite evolution in the first layer of austenitic stainless steel (ASS) 308L produced by WAAM-GTAW. Case Stud. Therm. Eng..

[34] Niu F., Bi W., Zhang K., Sun X., Ma G., Wu D. (2023). Additive manufacturing of 304 stainless steel integrated component by hybrid WAAM and LDED. Mater. Today Commun..

[35] Andrade D.G., Tankova T., Zhu C., Silva L.S., Rodrigues D.M. (2024). Mechanical properties of 3D printed CMT-WAAM 316LSi stainless steel walls. J. Constr. Steel Res..

[36] Vinoth V., Sathiyamurthy S., Natarajan U., Venkatkumar D., Prabhakaran J., Prakash S.K. (2022). Examination of microstructure properties of AISI 316L stainless steel fabricated by wire arc additive manufacturing. Mater. Proc..

[37] Chen W., Guo S., Xuan Y., Xu J., Li S., Fang W., Zhou Q., Wang K. (2024). Influence of different deposition strategies on the microstructure and mechanical properties of the laminated heterostructured material with ER130S-G HSS and 316 L SS fabricated by WAAM. Mater. Charact..

[38] Rezende R.F., Árias A.R., Lima E.J., Coelho F.G.F. (2025). Pulsed GMAW-based WAAM–Influence of droplet detachment mode on the geometry and mechanical properties of 308 L stainless steel. J. Adv. Join. Process..

[39] Yadav A., Srivastava M., Jain P.K., Rathee S. (2024). Microstructure transformations and improving wear resistance of austenitic stainless steel additively fabricated by arc-based DED process. Def. Technol..

[40] Chen Y., Zhao X., Yang B., Liu Y., Liang Y., Li Z., Chen C. (2024). Study on properties of 304 wire arc additive manufacturing stainless steel TIG welded joints. Mater. Lett..

[41] Sandeep O.S., Kuriachen B., Anantharam G.S., Eldose K.K. (2025). Influence of microstructure on the anisotropic tribocorrosion behaviour of WAAM-fabricated SS304L in marine environments. Tribol. Int..

[42] Wang L., Xue J., Wang Q. (2019). Correlation between arc mode, microstructure, and mechanical properties during wire arc additive manufacturing of 316L stainless steel. Mater. Sci. Eng. A.

[43] Ayan Y., Kahraman N. (2024). Investigation of tensile and fatigue properties of an austenitic stainless steel part fabricated by WAAM. Mater. Chem. Phys..

[44] Yao P., Lin H., Wu W., Tang H. (2021). Influence of Duty Ratio and Current Mode on Robot 316L Stainless Steel Arc Additive Manufacturing. Metals.

[45] Xiao X., Yin Y., Li C., LI C., Zhang K. (2020). Influence of thermal behavior on microstructure evolution of TIG arc additive manufacture for 316L stainless steel. Trans. Mater. Heat. Treat..

[46] Zhang H., Huang J., Liu C., Ma Y., Han Y., Xu T., Lu J., Fang H. (2020). Fabricating Pyramidal Lattice Structures of 304 L Stainless Steel by Wire Arc Additive Manufacturing. Materials.

[47] Guan K., Wang Z., Gao M., Li X., Zeng X. (2013). Effects of processing parameters on tensile properties of selective laser melted 304 stainless steel. Materials.

[48] Zhang D., Prasad A., Bermingham M.J., Todaro C.J., Benoit M.J., Patel M.N., Qiu D., StJohn D., Qian M., Easton M.A. (2020). Grain Refinement of Alloys in Fusion-Based Additive Manufacturing Processes. Metall. Mater. Trans..

[49] Liao H., Kuang Y., Zhou D., Zhang Q., Wu X., Xu L., Wang X., Wang Z. (2024). Achieving fine-grained structure with larger ferrite fraction via local dry underwater WAAM to enhance corrosion behavior of 308 L stainless steel. Constr. Build. Mater..

[50] Spinasa A., Weber B., Meng X., Zhang R., Nitawaki M., Gardner L. (2025). Influence of process parameters on the physical and material properties of WAAM steels. Constr. Build. Mater..

[51] Belhadj M., Werda S., Kromer R., Darnis P. (2025). Influence of operating parameters on the mechanical and geometric properties of 316L Stainless Steel structures fabricated by WAAM-CMT. Procedia CIRP.

[52] Wang F., Chen W., Wang D., Hou H., Zhao Y. (2024). Phase-field modeling and Experimental investigation for rapid solidification in wire and arc additive manufacturing. J. Mater. Res. Technol..

[53] Gurol U., Kocaman E., Dilibal S., Koçak M. (2023). A comparative study on the microstructure, mechanical properties, wear and corrosion behaviors of SS 316 austenitic stainless steels manufactured by casting and WAAM Technologies. CIRP J. Manuf. Sci. Technol..

[54] Bertsch K.M., Bellefon G.M., Kuehl B., Thoma D.J. (2020). Origin of dislocation structures in an additively manufactured austenitic stainless steel 316L. Acta Mater..

[55] Wang G., Ouyang H., Fan C., Guo Q., Li Z., Yan W., Li Z. (2020). The origino f high-density dislocations in additively manufactured metals. Mater. Res. Lett..

[56] Derekar K.S. (2018). A review of wire arc additive manufacturing and advances in wire arc additive manufacturing of aluminium. Mater. Sci. Technol..

[57] Mendez-Morales M., Jesus J.S., Branco R., Tankova T., Rebelo C. (2025). Fatigue crack growth of untreated and heat-treated WAAM ER70S-6 carbon steel. Int. J. Fatigue.

[58] Kun L., Ping Y., Sindo K. (2020). Solidification Cracking Susceptibility of Stainless Steels: New Test and Explanation. Weld. Res..

[59] Zhang C., Gao M., Zeng X. (2019). Workpiece vibration augmented wire arc additive manufacturing of high strength aluminum alloy. J. Mater. Process. Technol..

[60] Siddiqui N.A., Muzamil M., Jamil T., Hussain G. (2025). Heat sources in wire arc additive manufacturing and their impact on macro-microstructural characteristics and mechanical properties—An overview. Smart Mater. Manuf..

[61] Vimal K.E.K., Srinivas M.N., Rajak S. (2021). Wire arc additive manufacturing of aluminium alloys: A review. Mater. Today Proc..

[62] Strano G., Hao L., Everson R.M., Evans K.E. (2013). Surface roughness analysis, modelling and prediction in selective laser melting. J. Mater. Process. Technol..

[63] Rahimi A., Yazdizadeh M., Ara M.V., Pouranvari M. (2025). Strength and ductility of additively manufactured 310 austenitic stainless steel via wire-arc directed energy deposition: The role of columnar grain growth and ductility-dip cracking. Mater. Sci. Eng. A.

[64] (2024). Standard Test Methods for Tension Testing of Metallic Materials.

[65] Xiong J., Tzempelikos A., Bilionis I., Awalgaonkar N., Lee S., Konstantzos I., Sadeghi S., Karava P. (2018). Inferring personalized visual satisfaction profiles in daylit offices from comparative preferences using a Bayesian approach. Build. Environ..

[66] Felice I., Shen J., Barragan A.F., Moura I.A., Li B., Wang B., Khodaverdi H., Mohri M., Schell N., Ghafoori E. (2023). Wire and arc additive manufacturing of Fe-based shape memory alloys: Microstructure, mechanical and functional behavior. Mater. Des..

[67] Alagha A., Hussain S., Zaki W. (2021). Additive manufacturing of shape memory alloys: A review with emphasis on powder bed systems. Mater. Des..

[68] Ji L., Lu J., Liua C., Jing C., Fan H., Ma S. Microstructure and mechanical properties of 304L steel fabricated by arc additive manufacturing. Proceedings of the 2017 International Conference on Electronic Information Technology and Computer Engineering (EITCE 2017).

[69] Sasikumar C., Oyyaravelu R. (2024). Mechanical properties and microstructure of SS 316 L created by WAAM based on GMAW. Mater. Commun..

[70] Long P., Wen D., Min J., Zheng Z., Li J., Liu Y. (2021). Microstructure Evolution and Mechanical Properties of a Wire-Arc Additive Manufactured Austenitic Stainless Steel: Effect of Processing Parameter. Materials.

[71] Omiyale B., Ogedengbe I., Olugbade T., Farayibi P. (2024). Corrosion Performance of Wire Arc Additive Manufacturing of Stainless Steel: A Brief Critical Assessment. 3D Print. Addit. Manuf..

[72] de Brito Neto F.M., Ferreira M.O.A., dos Santos S.A.C., Pereira J.N., Meza D.L.C., Ahmed W., Nossa T.d.S., Moreto J.A., Pinto H.C., Arantes V.L. (2025). Mechanical and corrosion behaviour of 316L stainless steel processed by wire arc additive manufacturing (WAAM). Prog. Addit. Manuf..

[73] Chen M., Gong Z., Zhang T., Zuo W., Zhao Y., Zhao O., Zhang G., Wang Z. (2024). Mechanical behavior of austenitic stainless steels produced by wire arc additive manufacturing. Thin-Walled Struct..

[74] Nagasai B., Malarvizhi S., Balasubramanian V. (2022). Mechanical Properties and microstructural characteristics of wire arc aditive manufactured 308L stainless steel cylindrical componentes made by gas metal arc and colda metal transfer arc welding processes. J. Mater. Process. Technol..

[75] Karlina A.I., Kondratyev V.V., Balanovskiy A.E., Astafyeva N.A., Yamshchikova E.A. (2024). Porosity reduction in metal with hybrid wire and arc additive manufacturing technology (WAAM). CIS Iron and Steel Review..

[76] Jin W., Zhang C., Jin S., Tian Y., Wellmann D., Liu W. (2020). Wire Arc Additive Manufacturing os Stainless Steels: A Review. Materials.

[77] Keller T., Lindwall G., Ghosh S., Ma L., Lane B.M., Zhang F., Kattner U.R., Lass E.A., Heigel J.C., Idell Y. (2017). Application of Finite Element, Phase-field, and CALPHAD-based Methods to Additive Manufacturing of Ni-based Superalloys. Mater. Sci..

[78] Nedjad S., Yildiz M., Saboori A. (2023). Solidification behaviour os austenitic stainless steels during welding and directed energy deposition. Sci. Technol. Weld. Join..

[79] Kotecki D.J., Lippold J.C. (2005). Welding Metallurgy and Weldability of Stainless Steels.

[80] Ettefagh A.H., Guo S., Raush J. (2021). Corrosion performance of additively manufactured stainless steel parts: A review. Addit. Manuf..

[81] Charmi A., Falkenberg R., Ávila L., Mohr G., Sommer K., Ulbricht A., Sprengel M., Neumann R.S., Skrotzki B., Evans A. (2021). Mechanical anisotropy of additively manufactured stainless steel 316L: An experimental and numerical study. Mater. Sci. Eng. A.

[82] Wang H., Jiang P., Yang G., Yan Y. (2024). An Investigation of the Anisotropic Mechanical Properties of Additive-Manufactured 316L SS with SLM. Materials.

[83] Wang C., Liu T.G., Zhu P., Lu Y.H., Shoji T. (2020). Study on microstructure and tensile properties of 316L stainless steel fabricated by CMT wire and arc additive manufacturing. Mater. Sci. Eng. A.

[84] Ajay V., Nakrani J., Mishra N.K., Shrivastava A. (2023). Anisotropic fatigue crack propagation in wire arc additively manufactured 316L stainless steel. Int. J. Fatigue.

[85] Becker T.H., Kumar P., Ramamurty U. (2021). Fracture and fatigue in additively manufactured metals. Acta Mater..

[86] Rodriguez N., Vázquez L., Huarte I., Arruti E., Tabernero I., Alvarez P. (2018). Wire and arc additive manufacturing: A comparison between CMT and TopTIG processes applied to stainless steel. Weld. World.

[87] Laghi V., Palermo M., Tonelli L., Gasparini G., Ceschini L., Trombetti T. (2020). Tensile properties and microstructural features of 304L austenitic stainless steel produced by wire-and-arc additive manufacturing. Int. J. Adv. Manuf. Technol..

[88] (2013). Standard Specification for Stainless Steel Bars and Shapes.

[89] Gowthaman P.S., Jeyakumar S., Sarathchandra D.T. (2023). Experimental characteristics of 316L stainless steel thin wall processed in wire-based arc additive manufacturing process. Inst. Mech. Eng..

[90] Anand S., Haldar N., Datta S., Das A. (2023). Experimental investigation on microstructure and mechanical property of wire arc additively manufactured SS308L built part. Sadhana.

[91] Rani K.U., Kumar R., Mahapatra M.M., Mulik R.S., Swierczynska A., Fydrych D., Pandey C. (2022). Wire Arc Additive Manufactured Mild Steel and Austenitic Stainless Steel Components: Microstructure, Mechanical Properties and Residual Stresses. Materials.

[92] (1990). ASM Handbook, Volume 1. Properties and Selection: Irons, Steels, and High Performance Alloys Section: Publication Information and Contributors.

